# A practical approach for the stable isolation and cultivation of chicken gonadal primordial germ cells with mitotically inactivated STO feeder cells

**DOI:** 10.5713/ab.25.0192

**Published:** 2025-06-04

**Authors:** Hyeon Yang, Bo Ram Lee, Jae-Yeong Lee, Keon Bong Oh, Poongyeon Lee, Seunghoon Lee, Yong Jin Jo, Haesun Lee, Seokho Kim, Jingu No, Jae Yong Han, Sung June Byun

**Affiliations:** 1 Animal Biotechnology Division, National Institute of Animal Science, Rural Development Administration, Wanju, Korea; 2 Department of Agricultural Biotechnology and Research Institute of Agriculture and Life Sciences, College of Agriculture and Life Sciences, Seoul National University, Seoul, Korea; 3 Poultry Research Institute, National Institute of Animal Science, Rural Development Administration, Pyeongchang, Korea

**Keywords:** Chicken, Feeder Cells, Gonadal Primordial Germ Cells, Stable Cultivation, Transgenic

## Abstract

**Objective:**

Establishing chicken primordial germ cells (PGCs) *in vitro* is critical for producing genetically modified (GM) chickens. Efficient and reliable isolation and cultivation of PGCs remain significant challenges in advancing avian genetic modifications. To address these challenges, we employed a streamlined and practical approach for the efficient isolation and stable cultivation of chicken gonadal PGCs.

**Methods:**

Chicken gonadal PGCs were isolated from embryonic gonads, surgically removed and dissociated using trypsin. The PGCs were isolated by exploiting differential adhesion properties, allowing fibroblasts to attach while PGCs remained suspended. Cultivation was performed with mitotically inactivated SIM mouse embryo-derived thioguanine-resistant (STO) feeder cells under optimized culture conditions.

**Results:**

PGCs proliferated robustly, reaching over 10^5^ cells within one month, which is comparable to previously reported methods. Characterization assays confirmed the expression of PGC-specific markers, including SSEA-1 and DAZL, along with pluripotency-related genes such as *OCT4* and *NANOG*. Additionally, injected PGCs successfully migrated to recipient embryonic gonads, where their presence was confirmed by fluorescence analysis and PCR.

**Conclusion:**

This study highlights the effectiveness of the STO feeder-based culture system in avian germ cell research, contributing to progress in the production of germline chimeric and GM chickens.

## INTRODUCTION

Primordial germ cells (PGCs), the precursors of gametes, are essential for avian reproduction and the development of genetically modified (GM) chickens. Their capacity for stable long-term proliferation and genetic modification *in vitro* has enabled significant progress in developmental biology, agriculture, and biomedicine [1].

Recent advancements in genome editing technologies, particularly the clustered regularly interspaced short palindromic repeats (CRISPR)/CRISPR-associated protein 9 (Cas9) system, have significantly enhanced the utility of chicken PGCs in avian biotechnology [2]. This system enables precise, site-specific genome editing in chicken PGCs, allowing targeted modifications at a specific locus. When applied to PGCs, the CRISPR/Cas9 system offers practical advantages such as procedural simplicity, high editing efficiency, and reduced cytotoxicity, making it a preferred tool for avian genome engineering [3]. Several studies have demonstrated the effective use of this strategy to produce gene-edited chickens, including models with resistance to avian influenza through modification of the *ANP32* gene family, and lines producing humanized antibodies [4,5].

Despite these advances, practical handling of PGCs remains technically demanding. Conventional methods for PGCs isolation such as cell sorting or density gradient centrifugation require specialized instruments and technical expertise, making them less accessible for routine applications [6–8]. Moreover, the limited number of PGCs that can be obtained from individual embryos imposes an additional limitation for downstream applications [9,10]. In addition, extended *in vitro* culture often involves complex protocols, including frequent passaging and supplementation with various growth factors, which could potentially affect the consistency and reproducibility of the culture outcomes [11,12]. These limitations indicate the necessity of a simplified, technically accessible, and reproducible protocol that enables stable proliferation and maintenance of PGC identity under manageable laboratory conditions.

In this study, we aimed to establish a practical and accessible system for chicken PGC culture that overcomes the limitations of conventional methods. Our approach emphasizes reduced technical complexity while maintaining stable proliferation and key germline characteristics. This provides a reproducible framework for avian germ cell research and the generation of GM chickens.

## MATERIALS AND METHODS

### Animal care and use

Fertile eggs for the derivation of wild-type (WT) PGCs were produced via artificial insemination using semen collected from WT White Leghorn (WL) male chickens and WT WL female chickens. Additionally, heterotypic transgenic males expressing the 3D8 single-chain variable fragment (scFv) gene [13] and WT WL female chickens were paired to generate fertile eggs carrying the 3D8 scFv transgene. To distinguish transgenic from WT embryos, embryonic blood was collected for polymerase chain reaction (PCR) screening. All embryos were continuously incubated after sampling, and only those identified as positive for the 3D8 scFv gene were used for the isolation and culture of transgenic PGCs. The primers used were: forward, CCT CTG CTA ACC ATG TTC ATG CCT TC; reverse, GCT AGT GAA TGT GTA TCC AGA AGC CTT. All poultry were housed and managed under standard conditions at the institutional poultry farm. The experimental procedures were approved by the Institutional Animal Care and Use Committee (IACUC) of the National Institute of Animal Science (NIAS-2023-593), Republic of Korea, in accordance with ethical guidelines for animal research.

### Isolation of gonadal primordial germ cells

Freshly laid fertilized eggs were incubated at 37.5°C until the embryos reached 5.5 to 6.0 days of development. The embryos were washed twice with phosphate-buffered saline (PBS; Gibco) and placed on black wax-coated Petri dishes. The embryonic gonads were surgically removed using sterilized tweezers and transferred to 1.5 mL microcentrifuge tubes containing 400 μL of PBS supplemented with 0.5% bovine serum albumin (BSA; Sigma-Aldrich). Subsequently, 100 μL of 0.25% trypsin-ethylenediaminetetraacetic acid (EDTA) was added (final concentration of 0.05%), and the samples were incubated at 37.5°C in 5% CO_2_ for 10–15 minutes to dissociate the tissue. The digested suspension was diluted with 4.5 mL of PBS and centrifuged at approximately 120×g for 5 minutes, after which the pellet was resuspended in PGC culture medium. The cell suspension was seeded into a 6-well culture plate, and after 4–5 hours, nonadherent cells were transferred to new 6-well plates precoated with mitotic inactivated feeder cells.

### Preparation of the feeder cells

The feeder cells used incubated SIM mouse embryo-derived thioguanine-resistant (STO) cells (CRL-1503), mouse embryonic fibroblast (3T3-L1) cells (CL-173), and Buffalo rat liver (BRL/3A) cells (CRL-1442), which were all purchased from the American Type Culture Collection (ATCC). The cells were cultured in Dulbecco’s modified Eagle’s medium (DMEM; Gibco) supplemented with 10% fetal bovine serum (FBS; Gibco) and 1× antibiotic-antimycotic (anti-anti; Gibco). Feeder cells were mitotically inactivated via treatment with 10 μg/mL mitomycin C (Roche) for 2 hours. The cells then washed thoroughly with PBS and detached using 0.05% trypsin-EDTA. Finally, inactivated feeder cells were frozen in Cell Banker Solution (AMSBIO) until use. For PGC culture, inactivated feeder cells were thawed and seeded 0.1% gelatin-coated 6-well culture plates.

### Cultivation of the primordial germ cells

The resuspended PGCs were cultured in knockout DMEM (KO-DMEM; Gibco) supplemented with 20% heat-inactivated FBS, 2% chicken serum (Gibco), 20% BRL/3A conditioned medium, 1× L-glutamine, 1× Minimum essential medium nonessential amino acids, 1× nucleotide mix, 1× antibiotic-antimycotic, and 1 mM sodium pyruvate. The BRL/3A cells for conditioned medium preparation were cultured in DMEM supplemented with 10% FBS and 1× anti-anti until approximately 90% confluence. The cells were then treated with mitomycin C, washed three times with PBS, and cryopreserved. For conditioned medium preparation, thawed cells were maintained in KO-DMEM for three days, and the collected supernatant was stored at −20°C, used at 20% (v/v) concentration. Growth factors, including 10 ng/mL basic fibroblast growth factor (bFGF), 10 ng/mL stem cell factor (SCF), 10 ng/mL insulin-like growth factor 1 (IGF-1), and 10 ng/mL activin A, were added to optimize the culture conditions. The cultures were maintained at 37.5°C in a 5% CO_2_ atmosphere, with media replaced every 2–3 days. The cells were monitored regularly, and PGCs were transferred to fresh wells when they reached confluence. Transgenic 3D8 PGCs were also cultured using this system under the same conditions as WT PGCs, following identical media composition, feeder preparation, and maintenance procedures.

### Immunofluorescence analysis

Primary isolated PGCs (denoted PGCs #1 and PGCs #2) were plated 4-well culture plates. The cells were fixed with 4% paraformaldehyde (Invitrogen) for 10 minutes and permeabilized using 0.5% Triton X-100 for 15 minutes at room temperature. After being blocked with 5% BSA, the cells were incubated overnight at 4°C with primary antibodies against stage-specific embryonic antigen-1 (SSEA-1; Thermo Scientific, 41-1200, 1:200) and deleted in azoospermia-like (DAZL; Thermo Scientific, BS-12245R, 1:200). Secondary antibodies were purchased from Abcam. Alexa Fluor 488-conjugated goat anti-mouse IgG (Abcam, ab150113, 1:500) and Alexa Fluor 647-conjugated goat anti-rabbit IgG (Abcam, ab150079, 1:500) were applied for 1 hour. Nuclei were counterstained with Hoechst 33258, and images were captured using a CoolLED’s pE-300-W microscope (Optinity, Korealabtech). To evaluate the early migration potential of cultured PGCs, the cells were labeled with the PKH67 Green Fluorescent Cell Linker Mini Kit (Merck) according to the manufacturer’s instructions. The labeling followed the standard PKH67 protocol, including dye incubation and quenching steps.

### Reverse transcription polymerase chain reaction analysis

Total RNA was extracted from gonadal tissues and PGCs using an RNeasy Mini Kit (Qiagen). Complementary DNA (cDNA) was synthesized using the Superscript IV First-Strand Synthesis System (Invitrogen). Reverse transcription polymerase chain reaction (RT–PCR) was performed to assess the expression of PGCs-specific markers, including *DAZL* and DEAD-box helicase 4 (*DDX4*), and pluripotency-related genes such as octamer-binding transcription factor 4 (*OCT4*), SRY-box transcription factor 2 (*SOX2*), and Nanog homeobox (*NANOG*), with glyceraldehyde-3-phosphate dehydrogenase (*GAPDH*) serving as the reference gene (Table 1). The primer sequences and PCR conditions were adopted from previously established protocols [14,15]. The amplified products were analyzed using agarose gel electrophoresis.

### Primordial germ cell microinjection

Recipient fertile eggs were incubated at 37.5°C until Hamburger and Hamilton stages 17–18 (2.5 days of development) [16]. For microinjection, eggs were briefly rotated by hand, and a 1.0–1.5 cm diameter hole was created at the pointed end of the shell. To assess the injection and incubation conditions, eggs were assigned to three experimental groups: (1) normal incubation without interventions, (2) incubation with an artificial hole at the pointed end, and (3) injection of DMEM into embryonic blood vessels through the artificial hole. PGCs were transferred with glass microcapillary needles (inner diameter: 45–55 μm) to inject 1–2 μL of KO-DMEM containing 1×10^3^ PGCs/μL into embryonic blood vessels. After injection, the hole was sealed with parafilm, and the eggs were incubated with the pointed end facing upward. Recipient embryos were maintained under standard incubation conditions until analysis.

### Lentivirus infection and selection

To investigate the migratory capacity of PGCs, lentivirus particles expressing enhanced green fluorescent protein (EGFP; hereafter referred to as GFP) were produced using a lentiviral vector system (VectorBuilder). The viral stocks contained 1.0–1.1×10^9^ transduction units/mL. The primary isolated PGCs (1×10^4^ cells/well) were seeded in 24-well gelatin-coated culture plates and transduced with lentivirus supplemented with 10 μg/mL polybrene for 24 hours at various multiplicities of infection (MOIs: 0, 1, 10, 100). Following transduction, the cells were cultured in medium containing 0.6 μg/mL puromycin for 6 days and then maintained under puromycin-free conditions for 14 days to allow for recovery. To enhance selection stringency, the cells were cultured for an additional 30 days with 0.3 μg/mL puromycin. Successfully transduced cells, designated GFP-PGCs, were used for further analysis, including characterization and migration assessment.

### Flow cytometry analysis

To quantify GFP expression, GFP-PGCs were harvested and washed twice with PBS. The cells were fixed with 4% paraformaldehyde for 5 minutes at room temperature and permeabilized with 0.1% Triton X-100 for 2 minutes. After washing twice with PBS, 1×10^4^ cells per sample were analyzed using a FACSCalibur flow cytometer (BD Bioscience).

### Statistical analysis

All the statistical analyses were conducted using GraphPad Prism 5.03 software (GraphPad). Differences in hatching rates among the experiment groups were evaluated using one-way ANOVA. A threshold of p<0.05 was considered to indicate statistical significance.

## RESULTS

### Establishment and functional validation of gonadal primordial germ cells

**Figure 1:**
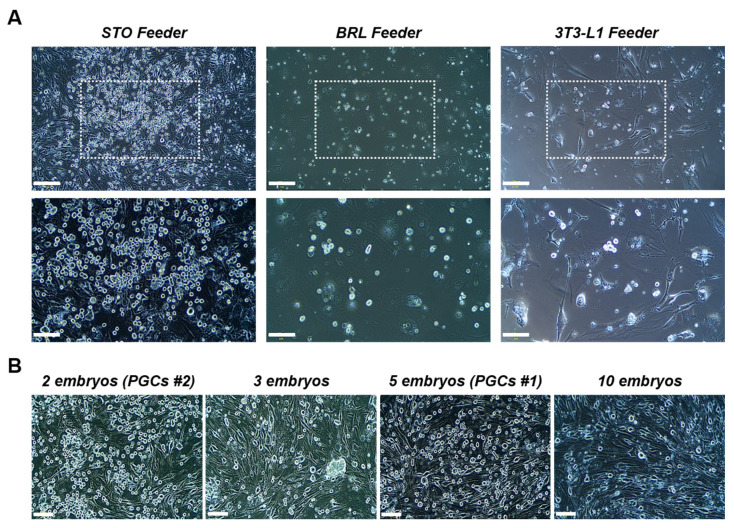
Establishment of gonadal primordial germ cells (PGCs) cultured with mitotically inactivated STO feeder cells. (A) Clusters of chicken gonadal PGCs cultured with mitotically inactivated SIM-derived thioguanine-resistant cells (STO), Buffalo rat liver (BRL/3A), and mouse embryonic fibroblast (3T3-L1) feeder cells and morphological analyses of the PGCs on each feeder type after 30 days of culture. Scale bars: 200 μm (upper) and 100 μm (lower). (B) Qualitative analyses confirming the number of gonadal PGCs according to the number of embryos used. The gonadal PGCs isolated from 2 embryos and 5 embryos, which presented with more than 10^5^ cells within a month, were denoted PGCs #1 (5 embryos) and PGCs #2 (2 embryos). Scale bars: 100 μm.

**Figure 2:**
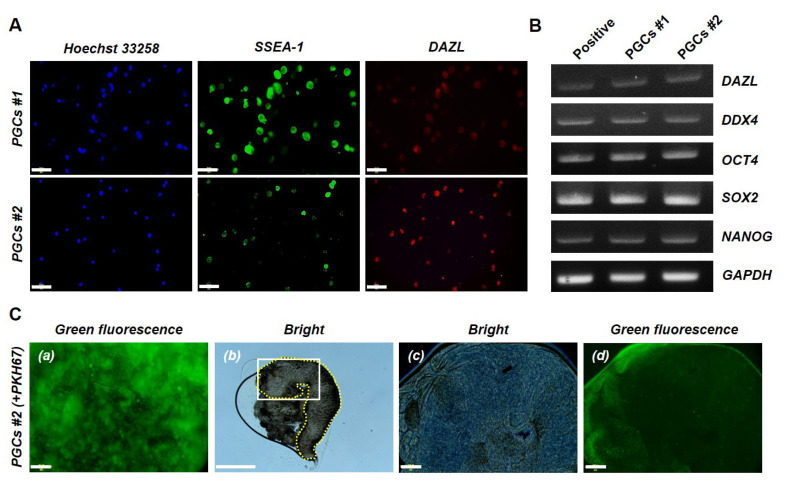
Characterization of gonadal primordial germ cells (PGCs) and validation using PKH67 labeling. (A) Stage-specific embryonic antigen-1 (SSEA-1) and deleted in azoospermia-like (DAZL) protein expression in the gonadal PGCs #1 and PGCs #2 was detected via immunofluorescence analyses. The cells were counterstained with Hoechst 33258. Scale bars: 50 μm. (B) Chicken *DAZL*, DEAD-box helicase 4 (*DDX4*), Octamer-binding transcription factor 4 (*OCT4*), SRY-box transcription factor 2 (*SOX2*), and Nanog homeobox (*NANOG*) mRNA expression in PGCs #1 and PGCs #2 was detected via reverse transcription polymerase chain reaction (RT–PCR). Chicken glyceraldehyde-3-phosphate dehydrogenase (*GAPDH*) mRNA expression was used as a PCR control. A complementary DNA stock from testes previously obtained from a newly hatched chick was used as a positive control. (C) PGCs #2 labeled using a PKH67 (green fluorescent dye) fluorescent cell linker kit were transferred to recipient embryos, and the green fluorescence in the embryonic gonad was detected at embryonic day 7. Scale bars: (a, c, d) 200 μm and (b) 500 μm.

The cell survival and propagation of PGCs isolated through enzymatic dissociation and differential adhesion-based selection were assessed. Morphological comparison was performed after 30 days of culture on each feeder type. Among the three feeder cell types tested, STO feeder cells demonstrated the highest efficiency in supporting PGC expansion, yielding greater cell proliferation and stability than BRL/3A and 3T3-L1 feeder cells did (Figure 1A). The number of embryos used for isolation did not appear to impact cell yield, as PGCs derived from five embryos (PGCs #1) and two embryos (PGCs #2) exhibited comparable proliferation rates, reaching approximately 10^5^ cells within one month (Figure 1B). Immunofluorescence staining confirmed the expression of SSEA-1 and DAZL, which was consistent with the PGC-specific marker profiles (Figure 2A). RT–PCR analysis further confirmed the expression of *DAZL*, *DDX4*, *OCT4*, *SOX2*, and *NANOG*, indicating the maintenance of both germline-specific and pluripotency-associated transcriptional signatures (Figure 2B). To assess the migratory capacity of cultivated PGCs, the PGCs #2 group was visualized by PKH67 labeling and injected into recipient embryos. Seven days after the transfer, fluorescence microscopy confirmed the presence of labeled PGCs in the developing gonads, demonstrating successful homing to the germline niche (Figure 2C). These findings collectively indicate that PGCs obtained via this approach exhibit stable expansion while preserving their molecular and functional properties, which are essential for germline transmission.

### Post-thaw proliferation of cryopreserved primordial germ cells

**Figure 3:**
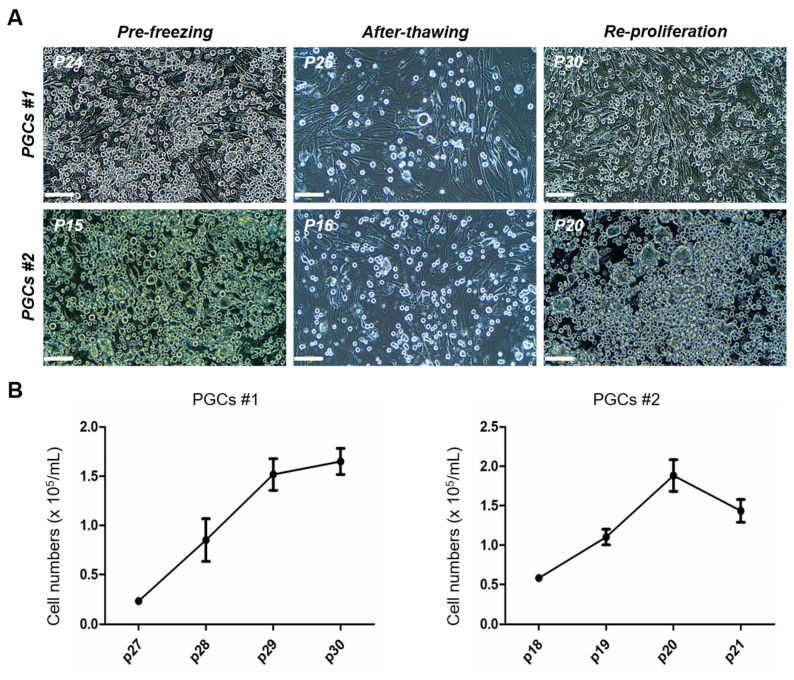
Proliferation of gonadal primordial germ cells (PGCs) after freezing and thawing. (A) Clusters of chicken gonadal GPCs (PGCs #1 and PGCs #2) before freezing (PGCs #1 at passage 24 and PGCs #2 at passage 15), immediately after thawing (PGCs #1 at passage 25 and PGCs #2 at passage 16), and after reproliferation (PGCs #1 at passage 30 and PGCs #2 at passage 20). Scale bars: 100 μm. (B) The number of gonadal PGCs from passages 27 to 30 for PGCs #1 and from passages 18 to 21 for PGCs #2. The number of gonadal PGCs was counted with a hemocytometer (n = 3).

To evaluate the proliferative ability of the PGCs following cryopreservation, PGCs #1 and PGCs #2 were frozen and thawed at passages 24 (p24) and 15 (p15), respectively. Both populations resumed proliferation after thawing, reaching cell counts exceeding 10^5^ (Figure 3A). The PGCs clusters remained intact, and continuous proliferation was observed from p25 to p30 for PGCs #1 and from p16 to p20 for PGCs #2 (Figure 3A). Quantitative analysis of cell numbers from p27 to p30 in PGCs #1 and p18 to p20 in PGCs #2 further confirmed their stable proliferation after cryopreservation (Figure 3B). These findings indicate that PGCs maintain their proliferative potential and viability after thawing, demonstrating their suitability for long-term preservation and subsequent applications.

### Generation and characterization of transgenic primordial germ cells

**Figure 4:**
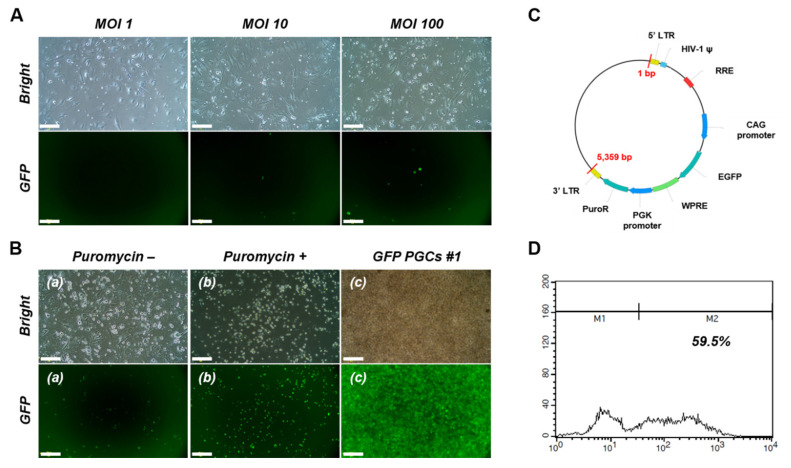
Construction of green fluorescent protein-primordial germ cells (GFP-PGCs) with a recombinant lentiviral vector. (A) Gonadal PGCs (PGCs #1) were seeded onto 24-well culture plates, and recombinant lentiviruses were added for 24 h at different multiplicities of infection (MOIs: 0, 1, 10, 100). PGCs were first screened for 6 days with puromycin (0.6 μg/mL). Scale bars: 200 μm. (B) PGCs first screened for 6 days were cultured for 14 days without puromycin. The PGCs were subsequently screened for 30 days with puromycin (0.3 μg/mL). Clusters of PGCs cultured without (−) or with (+) puromycin and recovered PGCs for transfer are shown (a, b, and c). Scale bar: (a, b) 200 μm and (c) 100 μm. (C) Vector map of a lentivirus expressing enhanced GFP. (D) Flow cytometry analysis showing PGCs with positive GFP signals (GFP-PGCs).

**Figure 5:**
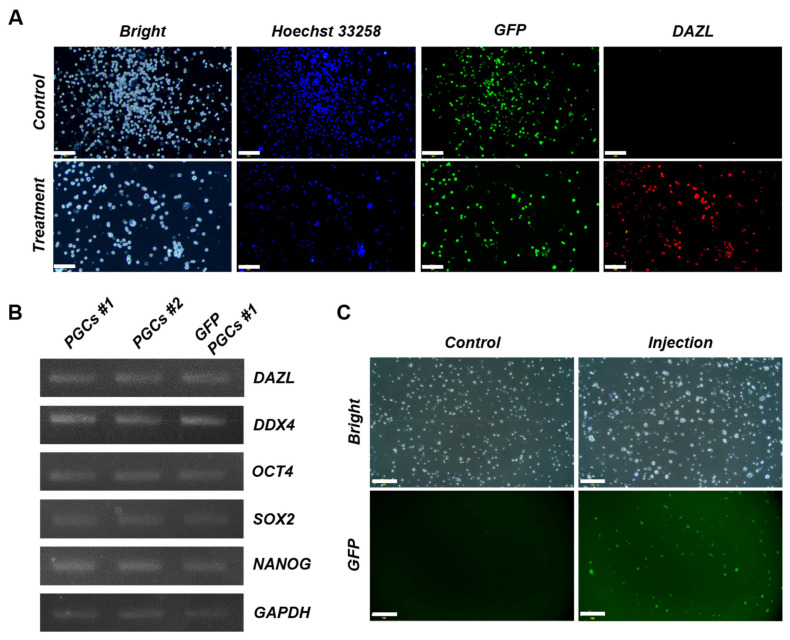
Characterization of green fluorescent protein-primordial germ cells (GFP-PGCs) and validation using GFP expression. (A) GFP and deleted in azoospermia-like (DAZL) protein expression in GFP-PGCs was confirmed via immunofluorescence. Treatment groups were stained with primary and secondary antibodies, while control groups were stained with secondary antibody only. The cells were counterstained with Hoechst 33258. Scale bars: 100 μm. (B) Chicken *DAZL*, DEAD-box helicase 4 (*DDX4*), octamer-binding transcription factor 4 (*OCT4*), SRY-box transcription factor 2 (*SRY2*), and Nanog homeobox (*NANOG*) mRNA expression in GFP-PGCs was detected via RT–PCR. Chicken glyceraldehyde-3-phosphate dehydrogenase (*GAPDH*) mRNA expression was used as a PCR control. (C) GFP expression in PGCs isolated from recipient embryos injected with GFP-PGCs, compared with control embryos without injection. Scale bars: 200 μm. RT–PCR, reverse transcription polymerase chain reaction.

To generate transgenic PGCs, PGCs #1 were transduced with a lentiviral vector carrying the GFP gene (Figure 4). GFP expression was first clearly detected at an MOI of 100 after the initial 6 days of puromycin selection (Figure 4A). The fluorescence signal persisted after 14 days of puromycin-free culture, and subsequent 30-day selection with 0.3 μg/mL puromycin resulted in a stable GFP-expressing population, designated GFP-PGCs (Figure 4B). Flow cytometry analysis confirmed that 59.5% of the GFP-PGCs expressed GFP (Figure 4D). Further characterization revealed that the germline competency of the GFP-PGCs was retained. Immunofluorescence and RT–PCR analysis confirmed the expression of PGC-specific markers and pluripotency-associated transcriptional profiles (Figures 5A, 5B). To assess *in vitro* functionality, GFP-PGCs were microinjected into recipient embryonic blood vessels, and three days posttransfer, GFP fluorescence was detected in primary cells isolated from recipient embryonic gonads, confirming successful migration and colonization within the germline niche (Figure 5C). These findings indicate that lentiviral-mediated transduction generated stable transgenic PGCs while preserving their germline characteristics and migratory potential.

### *In vivo* tracking of 3D8-transgenic primordial germ cells in recipient embryos

**Figure 6:**
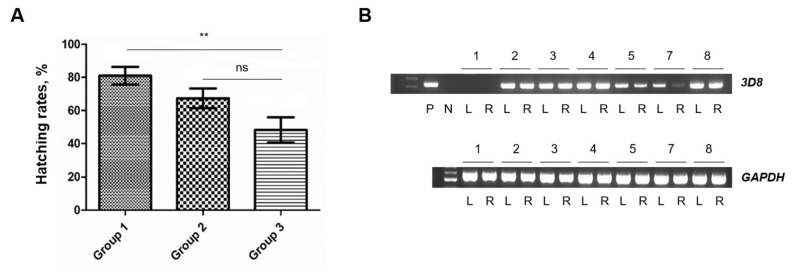
Validation of the transfer of 3D8 primordial germ cells (PGCs) into recipient embryonic gonads. (A) Hatching rates of fertile eggs in three groups: normal incubation without a hole (Group 1, 44 fertile eggs total), an artificial hole at point ends (Group 2, 54 total), and Dulbecco’s modified Eagle’s medium (DMEM) injection via the hole at point ends (Group 3, 53 total). The artificial hole was sealed with parafilm, and the sealed eggs were incubated and positioned point-end up (Group 2 and Group 3), after which the hatching rates of the groups were evaluated. The results are expressed as the mean±standard deviations (SDs) (n = 7). ** p<0.01. (B) Validation of the transfer of 3D8 PGCs through genomic DNA PCR. Each recipient is denoted with an Arabic numeral. P, positive; N, negative; L, left gonad; R, right gonad; PCR, polymerase chain reaction.

To evaluate the feasibility of PGC transplantation, the hatching rate of embryos following microinjection with DMEM was assessed. Among the injected eggs (Group 3), 48.3% successfully hatched, confirming that microinjection does not critically impair embryo viability compared with that of eggs with only an artificial hole (Group 2), as no statistically significant difference was observed between the two groups (p>0.05) (Figure 6A). To verify whether our culture and transplantation protocol is applicable to transgenic PGCs, we used transgenic 3D8 PGCs, which were isolated and cultured from a 3D8 transgenic chicken. These PGCs express the 3D8 gene, and their transplantation into recipient embryos allowed us to validate the effectiveness of our method for isolating and culturing PGCs from transgenic chickens. At embryonic day 12.5 (E12.5), the gonads of developing embryos were collected for PCR analysis. The presence of the 3D8 scFv gene was successfully detected, confirming that the injected PGCs migrated to and colonized the recipient gonads (Figure 6B). These findings confirm that microinjection-based PGC transplantation supports embryo viability and enables stable germline integration of transgenic PGCs.

## DISCUSSION

In this study, we established a simplified and reproducible culture system for chicken PGCs that overcomes key limitations of conventional protocols. Traditional PGC culture methods often require complex isolation procedures, feeder cell preparation, and media supplementation strategies that limit accessibility and standardization [17–21]. Our approach integrates differential adhesion-based isolation with STO feeder cell culture to streamline the process while maintaining stable proliferation and germline-specific characteristics. While differential adhesion-based enrichment [22] and STO feeder support [1] have been individually utilized in cell culture systems, their integration for gonadal PGC culture in this study offers practical advantages in reproducibility and broader applicability.

The culture system enabled stable and substantial expansion of gonadal PGCs, with representative lines reaching approximately 10^5^ cells/mL within one month, as observed in PGCs #1 (derived from 5 embryos) and PGCs #2 (derived from 2 embryos). This growth rate has been used in previous studies as a criterion to identify viable PGC lines suitable for downstream applications such as cryopreservation or chimera production [23,24]. Although similar expansion may also be achievable through other protocols, our study demonstrated that such consistent proliferation could be achieved using a combination of differential adhesion-based isolation and STO feeder cell support. The differential adhesion strategy allowed selective enrichment of floating PGCs by removing adherent somatic cells without the need for specialized equipment. In parallel, STO feeder cells consistently provided a supportive niche that prompted proliferation and reduced culture variability. This dual approach helped eliminate technical hurdles commonly associated with PGCs handling by replacing complex cell-sorting or multi-step enrichment procedures with a simple and reproducible method, thus promoting procedural simplicity and reliable PGC expansion.

As part of our preliminary efforts, we evaluated different sources and methods for PGC isolation and culture. Although blood-derived PGCs were tested, they exhibited poor proliferation under our culture conditions and presented significant handling difficulties due to their small size and the limited surface area of the culture plates. We also attempted magnetic-activated cell sorting for gonadal PGC enrichment, but the results were suboptimal and lacked consistency (data not shown). Based on repeated trials, we established the current protocol combining differential adhesion-based enrichment and STO feeder cells, as alternative feeder types such as BRL/3A or 3T3-L1 cells yielded less favorable outcomes. In particular, 3T3-L1 feeders frequently produced excessive cell debris, which interfered with PGC observation and maintenance. By contrast, our final approach also supported expansion from a single embryo, with PGCs reaching approximately 10^5^ cells/mL within one month (data not shown). Collectively, these findings confirm the robustness and efficiency of our integrated system for supporting the reliable propagation of chicken PGCs. With this robust culture foundation established, we further examined the role of specific media components in sustaining PGC proliferation and maintaining their germline and pluripotency profiles.

The culture medium used in this study consisted of bFGF, activin A, SCF, IGF-1, and leukemia inhibitory factor, the latter supplied through BRL/3A-conditioned medium [1,14, 25,26]. While the individual roles of each component were not dissected in this study, which we recognize as a limitation, this combination enabled consistent cell survival and expansion. Among these factors, FGF signaling, particularly through the mitogen-activated protein kinase/extracellular signal-regulated kinase pathway, has been reported as essential for chicken PGC culture [27,28]. However, in our initial trials, supplementation with bFGF alone did not support robust proliferation. This difference may be attributed to the source of PGCs and the feeder cell type, as previous studies demonstrating FGF-dependent culture success primarily utilized blood-derived PGCs [27] or mouse embryonic fibroblast feeder cells [29]. In contrast, the combination of signaling factors used in our system, optimized for gonadal PGCs cultured on STO feeders, supported more consistent long-term proliferation and maintenance of cellular identity. PGCs cultured under these conditions consistently maintained their characteristic morphology and actively expressed key germline and pluripotency markers, SSEA-1, *DAZL*, *DDX4*, *OCT4*, *SOX2*, and *NANOG*, thereby confirming the preservation of their germline identity during long-term culture.

To assess the functional capacity of cultured PGCs, we conducted *in vivo* migration assays using both PKH67-labeled and GFP-expressing cells. In both cases, the PGCs were successfully detected in recipient gonads and primary cells, confirming their successful migration and subsequent settlement within the gonadal niche. Additionally, PCR analysis of embryos injected with 3D8 PGCs, derived from 3D8 scFv transgenic embryos, confirmed the presence of the 3D8 transgene in gonadal tissue at E12.5. The 3D8 PGCs were distinct from the fluorescently labeled WT PGCs, as they originated from transgenic chicken embryos rather than from WT donors. Notably, the detection of the 3D8 transgene in recipient gonads confirmed that even transgenic PGCs maintained their ability to migrate and colonize the gonadal tissue after *in vitro* culture and transfer. While our study did not extend to the production of G0 candidates or test crossing, which would provide definitive evidence of germline transmission, the results demonstrate the feasibility of using the established culture system for preparing functional PGCs. Therefore, this system offers a rapid and reliable platform for generating both WT and transgenic PGCs suitable for downstream applications in avian biotechnology.

## CONCLUSION

Taken together, the culture system presented in this study offers a simplified and reproducible approach for generating functional chicken PGCs. Its applicability to both WT and transgenic lines, along with the ability to support long-term proliferation and *in vivo* colonization, highlights its value for avian biotechnology, including genetic resource preservation and genome engineering. By integrating differential adhesion-based isolation with STO feeder support and a defined culture environment, this platform addresses key limitations of earlier protocols. This system serves as a practical platform for future efforts in germline modification and transgenic chicken production.

## Figures and Tables

**Table 1:** Primer information for reverse transcription polymerase chain reaction (RT–PCR) analysis

Gene	Sequences	References
*DAZL*	F: TCCCAGAGCCCACACAGATG R: AAGTGATGCGCCCTCCTCTC	Whyte et al [14]
*DDX4*	F: TCCATCTTTGCATGTTATCAGTCAGG R: AATCCCGCCCTGCTTGTATAACAG	Whyte et al [14]
*OCT4*	F: GGCTCAATGAGGCAGAGAAC R: GGACTGGGCTTCACACATTT	Whyte et al [14]
*SOX2*	F: GTGAACCAGAGGATGGACAGTTACG R: TGCGAGCTGGTCATGGAGTTG	Whyte et al [14]
*NANOG*	F: AGCAGACCTCTCCTTGACCA R: TTCCTTGTCCCACTCTCACC	Whyte et al [14]
*GAPDH*	F: CCTCTCTGGCAAAGTCCAAG R: CATCTGCCCATTTGATGTTG	Rengaraj et al [15]
